# Editorial for Special Issue ‘Engineering and Characterisation of Novel Nanomedicine Formulations, 2nd Edition’

**DOI:** 10.3390/pharmaceutics17060763

**Published:** 2025-06-10

**Authors:** Raquel Fernández-García, Francisco Bolás-Fernández, Ana Isabel Fraguas-Sánchez

**Affiliations:** 1 School of Pharmacy, University of Nottingham, University Park, Nottingham NG7 2RD, UK; 2 Department of Microbiology and Parasitology, Faculty of Pharmacy, Complutense University, 28040 Madrid, Spain; 3 Department of Pharmaceutics and Food Technology, Faculty of Pharmacy, Complutense University, 28040 Madrid, Spain; 4 Institute of Industrial Pharmacy, Complutense University, 28040 Madrid, Spain

Nanomedicine applies nanotechnology to revolutionise healthcare through the development of systems at the nanoscale (below 1000 nm) to enhance drug delivery. These nanosystems are designed not only to optimise drug release profiles, but also to solve critical pharmaceutical challenges by encapsulating poorly soluble drugs and precisely targeting specific tissues, thus significantly reducing their adverse effects. Nanosystems can also protect drugs from degradation, proving especially valuable for sensitive therapeutic agents like proteins and RNA, thus unlocking new treatment possibilities.

This Special Issue, titled ‘Engineering and Characterisation of Novel Nanomedicine Formulations, 2nd Edition’, showcases cutting-edge developments in the nanomedicine field. The Special Issue consists of 11 research articles and 2 review articles that present diverse and significant advances in this rapidly evolving field. The articles cover multiple nanosystems, including inorganic and lipid nanoparticles, micelles, nanoemulsions, dendrimers and nanocrystals.

Mebeverine hydrochloride is an antispasmodic agent used to regulate bowel movements and relax intestinal smooth muscle; however, its application in clinical practice is limited due to its specific side effects [1]. Stoyanova et al. (contribution 1) developed silver nanoparticles loaded with this therapeutic agent to treat gastrointestinal spasmodic disorders [2]. These nanoparticles were synthesised using AgNO_3_ under previously optimised conditions. This formulation proved promising compared to other commercially available drugs, although it showed a lower anti-inflammatory effect than free mebeverine hydrochloride.

As discussed by Fernández-García and Fraguas-Sánchez (contribution 2), pulmonary drug delivery has recently become a hot topic in pharmaceutical development to combat respiratory infections and lung cancer. One of the main challenges is successful drug deposition into the lungs, as the respiratory system tries to eliminate inhaled particles. In this context, nanomedicine has emerged as a useful strategy to engineer particles with prolonged pulmonary retention, as they can avoid clearance while reducing systemic drug distribution and, thus, adverse systemic effects. In this context Party et al. (contribution 3) developed a nanoparticle-based dry-powder inhaler as a strategy to enable ciprofloxacin to exert targeted antibacterial activity in the lungs. This system was characterised by large lung deposition and a suitable aerodynamic diameter, which allows for the potential treatment of patients with different respiratory infections. Khan et al. (contribution 4) explored the pulmonary delivery of nanostructured lipid carriers loaded with trans-resveratrol to treat lung cancer, which is the leading cause of cancer death worldwide [3]. They developed 24 formulations in which one solid lipid (Dynasan 116) was combined with four liquid lipids (Capryol 90, Lauroglycol 90, Miglyol 810 and Tributyrin) at different solid/liquid ratios and different drug concentrations. The optimised formulation was stable for six months and exhibited excellent aerosolisation performance when using an air-jet nebuliser. Encapsulating trans-resveratrol within lipid nanocarriers enabled sustained drug release, which was markedly visible after 3 h, where free-drug levels reached ~76% of the total drug concentration, while nanocarriers released ~37%.

Due to the recent global healthcare situation, lipid nanoparticles have been studied to determine their efficacy in combating severe acute respiratory syndrome coronavirus 2 (SARS-CoV-2), particularly in engineering vaccine platforms [4]. The manufacturing of lipid nanoparticles in SARS-CoV-2 mRNA vaccines has garnered attention, as these can preserve and deliver mRNA to specific cells with a low to moderate immunological response [5]. Their effectiveness has been confirmed, as multiple lipid-nanoparticle-based mRNA vaccines against SARS-CoV-2 have been approved by regulatory agencies. However, lipid nanoparticles tend to accumulate in the liver, meaning that only a fraction of each dose reaches the target, and their poor long-term storage stability remains an obstacle. Lipid nanoparticles primarily consist of lipid components, including ionisable lipids, helper lipids (phospholipids and cholesterol) and polyethylene glycol (PEG)-containing lipids. The latter has a significant effect on the physicochemical properties, stability and in vivo kinetics of the formulation [6]. In this context, Saraswat et al. (contribution 5) utilised polysorbate 80 as an alternative PEG-lipid to manufacture lipid nanoparticles for the SARS-CoV-2 vaccine. They prepared lipid nanoparticles with a lipid composition of (6Z,9Z,28Z,31Z)-heptatriacont-6,9,28,31-tetraene-19-yl 4-(dimethylamino)butanoate (DLin-MC3-DMA), distearoylphosphatidylcholine (DSPC), cholesterol and polysorbate 80. This formulation showed enhanced spleen tropism over liver accumulation, maintaining high mRNA encapsulation and activity after over six months of storage at −80 °C.

It is well known that using micelles is an excellent strategy to enhance the solubility of poorly soluble drugs [7]. D-α-tocopherol PEG 100 succinate (VitE-TPGS) is an amphiphilic molecule widely used in formulation, as it is capable of forming micelles in aqueous environments due to its low critical micelle concentration [8]. De Caro et al. (contribution 6) used VitE-TPGS to prepare micelles in the presence and absence of poorly soluble pharmaceutical compounds. These micelles were then investigated in terms of their morphology to assess their ability to solubilise poorly soluble pharmaceutical compounds. For this purpose, a novel theoretical approach to analysing the pair distribution function was developed. This model was able to estimate the relative fractions of drugs incorporated into the core and shell of the micelles.

Stankovic et al. (contribution 7) developed intravenous nanoemulsions with a pyrazoloquionline ligand to enhance drug delivery to the brain. This ligand showed a unique profile of selective binding to σ2 receptors; however, its poor solubility hampers its research and development. An in vitro blood–brain barrier study demonstrated that a substantial part of the ligand, when applied in a nanoemulsion, was able to permeate the barrier.

It should be noted that nanomedicine can also be useful in regenerative medicine and tissue engineering. Extracellular vesicles (EVs) are nanosized particles secreted by various cell types that mediate intercellular communication. Galbiati et al. (contribution 8) used fibroblast-derived EVs to functionalise hydrogels. These hydrogels consisted of gelatin methacrylate (GelMA), which is a widely used biomaterial with tuneable mechanical, chemical and biological properties that is also able to support wound healing. In a murine model, hydrogels loaded with fibroblast-derived EVs significantly accelerated wound healing. These hydrogels, containing trehalose and polyvinylpyrrolidone 40 (PVP40), maintained a uniform population of vesicles, making them essential for preserving the structural and functional characteristics of EVs during freeze-drying.

Strzelecka et al. (contribution 9) synthesised a novel poly(amidoamine) (PAMAM) dendrimer/camptothecin complex as a novel strategy for targeted cancer treatment. These complexes controlled the release of camptothecin over long periods of time (>400 h).

Nanocrystals can be used in pharmaceutical research as a drug delivery system to improve the oral bioavailability of Biopharmaceutics Classification System (BCS) class II and IV drugs. For example, Cheng et al. (contribution 10) developed curcumin nanocrystal liposomes to overcome the limited bioavailability of this drug. The encapsulation of nanocrystals within liposomes showed significantly slower dissolution rates in simulated intestinal fluid than the nanocrystal alone, showing that the lipid bilayer impeded rapid core nanocrystal dissolution while showing superior mucus-penetrating capabilities in cell uptakes studies.

Micro- and nanomotors are devices with the ability to generate autonomous movement through the conversion of several forms of stimulation [9]. Among these stimuli, chemical reagents, light, ultrasound frequencies and magnetic fields can be found. Their ability to exert continuous movement enhances their interaction with target molecules, thus facilitating drug delivery, improving the accumulation rate in complex biological environments and enabling their penetration into tissues. Donoso-González et al. (contribution 11) developed a novel core–shell micromotor that combined magnetic and photothermal properties. It was synthesised via the template-assisted electrodeposition of iron and reduced graphene oxide on a microtubular pore-shaped membrane. The resulting iron-reduced graphene oxide core–shell micromotors, loaded with doxorubicin, successfully released the drug upon near-infrared irradiation.

Hybrid lipid nanoparticles that combine proteins with lipids represent an emerging class of nanocarriers designed to leverage the advantages of both biological and synthetic components for drug delivery, gene therapy and vaccine applications [10]. Maeyouf et al. (contribution 12) developed novel hybrid lipid nanoparticles that contained zein and were conjugated to the cancer-targeting ligand transferrin. Specifically, these nanoparticles were designed to entrap the anticancer drug docetaxel and carry plasmid DNA. This formulation efficiently entrapped docetaxel, leading to its increased uptake by different cancer cell lines and enhancing its anti-proliferative efficacy. Furthermore, proficient DNA condensation was demonstrated, resulting in increased gene expression across all tested cell lines.

Finally, a review article by Malheiro et al. (contribution 13) discussed the emergence of Pharma 4.0 technologies, such as additive manufacturing, machine learning, in silico modelling and digital twins, and their application in the manufacturing of non-biological complex drugs.
